# Environmental Origin of the Genus *Bordetella*

**DOI:** 10.3389/fmicb.2017.00028

**Published:** 2017-01-24

**Authors:** Illiassou Hamidou Soumana, Bodo Linz, Eric T. Harvill

**Affiliations:** ^1^Department of Infectious Diseases, University of GeorgiaAthens, GA, USA; ^2^Center for Vaccines and Immunology, University of GeorgiaAthens, GA, USA; ^3^Department of Veterinary and Biomedical Sciences, Pennsylvania State UniversityUniversity Park, PA, USA

**Keywords:** *Bordetella*, environmental strains, ecological niches, extra-host adaptation, environmental origin

## Abstract

Members of the genus *Bordetella* include human and animal pathogens that cause a variety of respiratory infections, including whooping cough in humans. Despite the long known ability to switch between a within-animal and an extra-host lifestyle under laboratory growth conditions, no extra-host niches of pathogenic *Bordetella* species have been defined. To better understand the distribution of *Bordetella* species in the environment, we probed the NCBI nucleotide database with the 16S ribosomal RNA (16S rRNA) gene sequences from pathogenic *Bordetella* species. Bacteria of the genus *Bordetella* were frequently found in soil, water, sediment, and plants. Phylogenetic analyses of their 16S rRNA gene sequences showed that *Bordetella* recovered from environmental samples are evolutionarily ancestral to animal-associated species. Sequences from environmental samples had a significantly higher genetic diversity, were located closer to the root of the phylogenetic tree and were present in all 10 identified sequence clades, while only four sequence clades possessed animal-associated species. The pathogenic bordetellae appear to have evolved from ancestors in soil and/or water. We show that, despite being animal-adapted pathogens, *Bordetella bronchiseptica*, and *Bordetella hinzii* have preserved the ability to grow and proliferate in soil. Our data implicate soil as a probable environmental origin of *Bordetella* species, including the animal-pathogenic lineages. Soil may further constitute an environmental niche, allowing for persistence and dissemination of the bacterial pathogens. Spread of pathogenic bordetellae from an environmental reservoir such as soil may potentially explain their wide distribution as well as frequent disease outbreaks that start without an obvious infectious source.

## Introduction

Bacteria of the genus *Bordetella* are of primary importance in human and veterinary medicine because of their ability to colonize the respiratory tract, causing a wide range of pulmonary and bronchial infections. The common human- and animal-adapted pathogens *B. pertussis, B. parapertussis*, and *B. bronchiseptica* are known as the “classical” *Bordetella* species. *B. pertussis* is a strictly human pathogen, but *B. parapertussis* consists of two lineages, one infecting humans and the other infecting sheep (Mattoo and Cherry, 2005). In contrast to these examples of adaptation to a single host, *B. bronchiseptica* colonizes a variety of animals and even humans (Register et al., 2015), resulting in a broad array of respiratory diseases, from asymptomatic colonization to lethal pneumonia (Goodnow, 1980). Phylogenetic analyses (Musser et al., 1986; Diavatopoulos et al., 2005) and genome comparisons (Parkhill et al., 2003) have revealed that *B. pertussis* and *B. parapertussis* represent human-adapted forms of *B. bronchiseptica* that have evolved independently from a *B. bronchiseptica*-like ancestor. The genus also contains a number of additional, more recently described species. For example, *B. avium* (Kersters et al., 1984) causes respiratory disease in birds. *B. hinzii* (Vandamme et al., 1995) is generally regarded as a non-pathogenic colonizer of the respiratory tract of poultry but some strains were shown to cause disease in turkeys when experimentally inoculated (Register and Kunkle, 2009). Meanwhile, the closely related species *B. pseudohinzii* colonizes laboratory mice (Ivanov et al., 2015, 2016). *B. holmesii* (Weyant et al., 1995) causes pertussis-like disease and septicemia in humans (Shepard et al., 2004), and *B. bronchialis, B. flabilis*, and *B. sputigena* (Vandamme et al., 2015) were also isolated from human respiratory specimens. In contrast to other bordetellae, *B. trematum* (Vandamme et al., 1996) and *B. ansorpii* (Ko et al., 2005) are not associated with respiratory problems but were isolated from human wound infection.

*B. petrii*, a species originally isolated from a dechlorinating bioreactor enriched by river sediment, represents the first described environmental species within the *Bordetella* genus (von Wintzingerode et al., 2001). *B. petrii* strains were also found in marine sponges (Wang et al., 2007), grass root consortia (Wang et al., 2007), and in other environmental samples as members of microbial communities involved in the degradation of aromatic hydrocarbons, such as benzenes (Bianchi et al., 2005; Wang et al., 2007). In apparent conflict with its environmental source, the *B. petrii* genome contains genes that allow for the synthesis and secretion of factors specifically associated with the virulence of the pathogenic *Bordetella* sp., including the BvgAS master regulon and filamentous hemagglutinin (Gross et al., 2008). In addition to these environmental sources, *B. petrii* was also isolated from immunocompromised patients with ear infection, cystic fibrosis and chronic pulmonary disease (Fry et al., 2005; Biederman et al., 2015; Nagata et al., 2015), suggesting broad adaptability of this bacterial species to both environmental conditions and as an opportunistic pathogen of humans and possibly other animals.

Other *Bordetella* species have been obtained from environments not associated with animal hosts. Ten different bacterial strains were cultured from cotton swabs taken from the plaster wall surface of 1300-year-old mural paintings inside the stone chamber of the Takamatsuzuka Tomb, an ancient circular burial mound in Japan. Taxonomic classification of these isolates revealed three novel species that were then named *B. muralis, B. tumulicola*, and *B. tumbae* (Tazato et al., 2015). Isolation of the bacteria from the paintings, but not from the surrounding stone walls, suggested that these species might be involved in the observed biodeterioration of the colorful paintings (Kigawa et al., 2013).

According to their 16S rRNA gene sequences, other environmental bacteria from soil also belong to the genus *Bordetella*. Interestingly, the majority of those samples originated from contaminated sites such as soil polluted with chlorinated benzenes (Wang et al., 2007), from arctic soils contaminated with polycyclic aromatic hydrocarbons such as oil, diesel fuel or tar (Eriksson et al., 2003), from the sediment of a municipal wastewater plant (Nisola et al., 2010) and from arsenic polluted soils (Cavalca et al., 2010; Bachate et al., 2012). All these observations suggest that members of the *Bordetella* genus may have the potential for biodegradation of a great variety of organic compounds.

Although these anecdotal findings suggest that members of the *Bordetella* genus may be found in nature, there is currently no systematic analysis of the occurrence of *Bordetella* outside human or animal hosts, and the potential impact of environmental isolates on human and animal health is uncertain. Environmental niches of pathogenic *Bordetella* species have been proposed but not identified. Yet, the ability of *Bordetella* to survive and persist outside mammalian hosts would allow for its greater dissemination and persistence, and could contribute to a wide distribution of infections and disease, often without an obvious infectious source.

Here, we search the NCBI nucleotide database for 16S ribosomal RNA gene sequences of *Bordetella*-like microorganisms from various environments and compare them to those of the described species, including the classical bordetellae, to determine their phylogenetic relatedness. We identified 10 clades of related strains, all of which contained samples isolated from environmental sources, though only four clades also contained sequences from animal-associated species. Sequences from environmental samples had a significantly higher genetic diversity and were located closer to the root of the phylogenetic tree than those from animal-associated isolates, suggesting an environmental origin of the genus *Bordetella*. In addition, we show that the animal-adapted pathogens *B. bronchiseptica* and *B. hinzii* grow efficiently in soil extract, indicating that diverse pathogenic bordetellae may have retained the ability to proliferate in the environment.

## Materials and methods

### Search for *Bordetella* 16S rRNA gene sequences in the NCBI nt database

The16S ribosomal gene sequences of *Bordetella hinzii* strain LMG 13501 (GenBank accession number NR_027537.1); *Bordetella holmesii* strain ATCC 51541 (NR_121717.1); and *Bordetella pertussis* strain Tohama I (AF142326.1) were each used as queries for BLAST search (blastn) against the NCBI nr/nt database using the default search parameters with a hitlist size of 5000. From the numerous hits, we excluded sequences of isolates from the known species that are mentioned in the introduction and selected only those that showed higher percentage of similarity to known *Bordetella* species than to bacteria from any other genus, including *Achromobacter*. As a control, we ran blastn searches with each of the identified sequences against the NCBI nr/nt database to remove potential false positives. The remaining sequences, all of which were from bacteria obtained from environmental sources, were considered for further analysis. All three searches using 16S rRNA sequences of *B. pertussis, B. hinzii*, and *B. holmesii* as queries, respectively, gave consistent results. The accession numbers were then explored for details on sample source, year of isolation, and associated publications. Most sequences were described as *Bordetella* sp. in the gene description, but some were designated as “uncultured bacterium.”

### Phylogenetic analysis and tree construction

All 16S rRNA sequences were aligned in Clustal Omega (http://www.ebi.ac.uk/Tools/msa/clustalo/), and the alignment was checked manually for consistency. Only sequences containing a 1376 bp gene fragment (near full-length) were used for further analyses. In order to identify the closely related species of environmental *Bordetella* isolates, the 16S ribosomal RNA gene sequences of members of 16 named *Bordetella* species were used as references; namely *B. pertussis* Tohama I, *B. parapertussis* BPP5, *B. bronchiseptica* RB50, *B. avium* 197N, *B. hinzii* LMG 13501, *B. pseudohinzii* 8-296-03, *B. holmesii* ATCC 51541, *B. trematum* DSM 11334, *B. ansorpii* SMC-8986, *B. bronchialis* LMG 28640, *B. sputigena* LMG 28641, *B. flabilis* LMG 28642, *B. petrii* Se-1111R, *B. muralis* T6220-3-2b, *B. tumulicola* T6517-1-4b, and *B. tumbae* T6713-1-3b (Table 1). The 16S rRNA gene sequences of *Burkholderia pseudomallei* NCTC13179 and *Ralstonia solanacearum* YP-01 were used as outgroups. The aligned and trimmed sequences (one per unique sequence) were used to generate a Neighbor-joining tree using the Maximum Composite Likelihood algorithm in Mega (Tamura et al., 2007), and bootstrap support was estimated running 100,000 replications. Nucleotide diversity (Π) within environmental samples and within animal-associated samples were estimated in DnaSP (Librado and Rozas, 2009), and 95% confidence limits (Π_95_) were estimated using an online confidence limit calculator (https://www.allto.co.uk/tools/statistic-calculators/confidence-interval-for-mean-calculator/).

**Table 1:** **Reference strains of named ***Bordetella*** species**.

**Strain name**	**References**	**GenBank accession number**	**16S rRNA sequence length (bp)**
*Burkholderia pseudomallei* NCTC 13179	Johnson et al., 2015	CP003976.1	1487
*Ralstonia solanacearum* YP-01	NCBI	FJ494776.1	1500
*Bordetella avium* 197N	Sebaihia et al., 2006	NR_074639.1	1487
*Bordetella bronchiseptica* RB50	Parkhill et al., 2003	BX640447.1	1487
*Bordetella hinzii* LMG 13501	Kattar et al., 2000	NR_027537.1	1487
*Bordetella parapertussis* BPP5	Park et al., 2012	HE965803.1	1489
*Bordetella holmesii* ATCC 51541	NCBI	NR_121717.1	1487
*Bordetella pertussis* Tohama I	Parkhill et al., 2003	AF142326.1	1487
*Bordetella trematum* DSM 11334	von Wintzingerode et al., 2001	NR_025404.1	1521
*Bordetella flabilis* LMG 28642	Vandamme et al., 2015	EU082162.1	1376
*Bordetella bronchialis* LMG 28640	Vandamme et al., 2015	EU082135.1	1416
*Bordetella sputigena* LMG 28641	Vandamme et al., 2015	KF601914.1	1376
*Bordetella ansorpii* SMC-8986	Ko et al., 2005	AY594190.1	1424
*Bordetella pseudohinzii* 8-296-03	Ivanov et al., 2016	JHEP02000033.1	1542
*Bordetella petrii* DSMZ12804	Gross et al., 2008	NC_010170	1487
*Bordetella muralis* T6220-3-2b	Tazato et al., 2015	LC053647.1	1456
*Bordetella tumbae* T6713-1-3b	Tazato et al., 2015	LC053656.1	1456
*Bordetella tumulicola* T6517-1-4b	Tazato et al., 2015	LC053650.1	1456

### Soil sample collection and *Bordetella* growth in soil extract

Soils were sampled in April 2016 at two random sites in State College, Pennsylvania, near a suburban park (40°48′40.7″ N 77°53′06.1″ W and 40°48′38.2″ N 77°53′04.2″ W). Each sample was collected to a depth of 20 cm and thoroughly mixed. Fifty grams of each soil sample (100 g total) was placed in a bottle which was filled to 500 ml with sterile PBS. The sample was homogenized by shaking for 10 min, then left to settle for 1 h at room temperature and carefully decanted. The soil-PBS suspension was filter sterilized. Single colonies of *B. bronchiseptica* strain RB50, *B. hinzii* strain L60, and *B. petrii* strain DSMZ12804 were picked from Bordet-Gengou (BG) agar (Difco) plates supplemented with 10% defibrinated Sheep's blood (HemoStat Laboratories, Dixon, CA, USA) and were cultured in liquid Stainer-Scholte medium (Stainer and Scholte, 1970) overnight at 37°C. The *Bordetella* inocula were prepared as follows. The cultures were centrifuged, resuspended in 1 ml PBS, and the optical density (OD_600_) was determined. Following five consecutive 10-fold dilutions in 1 ml PBS, 100 μl (= 10^6^-fold dilution) containing ~150 (*B. petrii*) or 240 bacterial cells (*B. hinzii* or *B. bronchiseptica*) were added to 5 ml of the soil extract resulting in starting concentrations of ~30 bacterial cells/ml (*B. petrii*) and 48 bacterial cells/ml (*B. hinzii, B. bronchiseptica*). Bacterial numbers were determined by plating an aliquot of each inoculum. The culture tubes were incubated at room temperature (25°C) with shaking. After 24, 48, and 72 h, 100 μl of each culture was plated on BG agar supplemented with 10% defibrinated sheep's blood to determine bacterial numbers. Each experiment was carried out in triplicate. The mean and ± standard error as well as analysis of variance (ANOVA) were conducted using Graphpad Prism version 6.04. The bacterial doubling time was calculated by the formula: doubling time = ln(2)/ln(*N*(*t*)/*N*(0))/*t*, where *N*(*t*) is the number of bacterial cells at time *t, N*(0) is the number of bacteria at time 0 and t is the time in hours.

## Results

### *Bordetella* in the environment

We mined the NCBI nucleotide databases for *Bordetella* spp. 16S rRNA gene sequences. The search resulted in a total of 71 *Bordetella* spp. 16S rRNA gene sequences (Table 2) in addition to those from the named species (Table 1) *B. bronchiseptica, B. parapertussis, B. pertussis, B. hinzii, B. pseudohinzii, B. holmesii, B. avium, B. trematum, B. ansorpii, B. flabilis, B. bronchealis, B. sputigena* (isolated from samples of human/animal origin), *B. petrii, B. tumbae, B. muralis*, and *B. tumulicola* (isolated from environmental samples). The corresponding strains were recovered from different environmental niches (Table 2), including soil (52 strains) and water (11 strains), and from 8 strains associated with plants. The soil samples were of diverse origin, including compost, cave rocks, and metal mines, but the majority were sampled at sites contaminated with oil and several halogenated cyclic hydrocarbons such as chlorinated benzenes or hexachlorocyclohexane. The samples from aquatic environments were also of diverse origin, namely industrial wastewater, a sulfur spring, lake water, surface sea water, and river biofilms. Several samples from plants were isolated from roots and thus at the plant-soil interface (Table 2). Thus, members of the genus *Bordetella* appear to be widespread across different environmental niches.

**Table 2:** *****Bordetella*** strains for which the 16S ribosomal RNA sequences were recovered from environmental samples**.

***Bordetella*** **strains**	**Isolation source**	**Country**	**References**	**GenBank accession No**.	**Sequence length (bp)**	**Duplicated sequences**
**SOIL ORIGIN**						
***Bordetella*** **sp. F2**	Chlorinated benzenes polluted soil	Germany	Wang et al., 2007	DQ453689.1	1527	4
***Bordetella*** **sp. E3**	Chlorinated benzenes polluted soil	Germany	Wang et al., 2007	DQ453688.1	1527	4
***Bordetella*** **sp. QJ2–5**	Chlorinated benzenes polluted soil	China	NCBI	DQ152013.1	1393	4
***Bordetella*** **sp. 2b05**	HCH-contaminated soil	India	NCBI	JF979304.1	1523	
***Bordetella*** **sp. 2f06**	HCH-contaminated soil	India	NCBI	JF979347.1	1523	
***Bordetella*** **sp. 2e11**	HCH-contaminated soil	India	NCBI	JF979341.1	1522	
***Bordetella*** **sp. 1h08**	HCH-contaminated soil	India	NCBI	JF979288.1	1519	
***Bordetella*** **sp. 1c11**	HCH-contaminated soil	India	NCBI	JF979241.1	1521	
***Bordetella*** **sp. 2c11**	HCH-contaminated soil	India	NCBI	JF979320.1	1523	
***Bordetella*** **sp. 2a09**	HCH-contaminated soil	India	NCBI	JF979298.1	1519	
***Bordetella*** **sp. ud1**	1,2,4-TCB contaminated soil	Germany	NCBI	FJ529833.1	1523	4
***Bordetella*** **sp. ud29**	1,2,4-TCB contaminated soil	Germany	NCBI	FJ529848.1	1523	
***Bordetella*** **sp. ud3b**	1,2,4-TCB contaminated soil	Germany	NCBI	FJ529835.1	1523	4
***Bordetella*** **sp. ud13a**	1,2,4-TCB contaminated soil	Germany	NCBI	FJ529840.1	1525	
***Bordetella*** **sp. IITR02**	1,2,4-TCB contaminated soil	India	NCBI	EU752498.1	1422	
***Bordetella*** **sp. CTN-10**	Chemical factory soil	China	NCBI	FJ598334.1	1398	2
***Bordetella*** **sp. 2–12**	Chemical factory soil	China	NCBI	FJ598328.1	1410	2
***Bordetella*** **sp. CTN-16**	Chemical factory soil	China	NCBI	FJ598326.1	1412	4
***Bordetella*** **sp. C16-Siri112**	Oil-contaminated soil	Iran	NCBI	JX500276.1	1397	5
***Bordetella*** **sp. p23(2011)**	Magnetite drainage, Iron mine	China	NCBI	HQ652588.1	1518	
***Bordetella*** **sp. e3(2011)**	Magnetite drainage, Iron mine	China	NCBI	HQ652587.1	1501	3
***Bordetella*** **sp. d16(2011)**	Magnetite drainage, Iron mine	China	NCBI	HQ652589.1	1507	3
***Bordetella*** **sp. f17(2011)**	Magnetite drainage, Iron mine	China	NCBI	HQ652590.1	1520	
***Bordetella*** **sp. FB-8**	Creek sediment from former uranium-mining area	Germany	NCBI	JN885794.1	1385	
***Bordetella*** **sp. A2–436**	Uranium mine	Portugal	NCBI	KF441609.1	1528	
***Bordetella*** **sp. J4**	Acid mine drainage	France	Delavat et al., 2013	HF568988.1	1410	
***Bordetella*** **sp. BAB-4396**	Soil	India	NCBI	KM289182.1	1499	
***Bordetella*** **sp. B4**	Paddy field by yellow river	China	NCBI	EU140499.1	1523	
***Bordetella*** **sp. MCYF11**	Lake Taihu sediment	China	Yang et al., 2014	KC734882.1	1385	
***Bordetella*** **sp. PTG4–17**	Sediment of the Indian ocean	India	NCBI	EU603444.1	1496	
***Bordetella*** **sp. RCC3**	Caves rock	India	NCBI	KC119149.1	1476	
***Bordetella*** **sp. RCC4**	Caves rock	India	NCBI	KC119150.1	1464	
***Bordetella*** **sp. M1–6**	Compost	China	Kato et al., 2004	AB039335.1	1531	
***Bordetella*** **sp. FS1413**	Compost	Finland	Partanen et al., 2010	FN667145.1	1464	
***Bordetella*** **sp. SMG22**	Compost	China	Guo et al., 2015	AM930282.1	1491	6
***Bordetella*** **sp. OT-2-E7**	Compost	China	Tian et al., 2013	JQ337611.1	1397	6
*Bordetella* sp. strain 2ABA4	Solid waste dumpsites	Nigeria	Sanuth et al., 2013	HE858274.1	1168	
*Bordetella* sp. OS17	Benten-Cho station soil	Japan	Matsumura et al., 2009	AB453298.1	980	
*Bordetella* sp. VVAR	Soil	Japan	NCBI	FJ588707.1	1451	
*Bordetella* sp. Ds-4	Cultivated soil	India	NCBI	HQ857791.1	727	
*Bordetella* sp. R-8	Garden soil	India	NCBI	JX130378.1	1319	
*Bordetella* sp. SPB-24	Garden soil	India	Bachate et al., 2012	JN208922.1	1403	
*Bordetella* sp. As3–3	Arsenic contaminated soil	Italy	Cavalca et al., 2010	FN392624.2	544	
*Bordetella* sp. AGO-03	Arsenic contaminated rice field	India	NCBI	AB696982.1	979	
*Bordetella* sp. ADP-18	Arsenic contaminated rice fields	India	NCBI	AB697485.1	674	
*Bordetella* sp. C16-Siri108	Oil-contaminated soil	Iran	NCBI	JX500272.1	1069	
*Bordetella* sp. C16-Siri113	Oil-contaminated soil	Iran	NCBI	JX500277.1	1295	
*Bordetella* sp. BF07B02	Agricultural soil	Burkina Faso	Colinon et al., 2013	KC195878.1	1381	
*Bordetella* sp. HPC772	Activated sludge of an effluent treatment plant	India	NCBI	AY838357.1	580	
*Bordetella* sp. PH21	Phenolic compounds-contaminated sediment	China	NCBI	JN171686.1	721	
*Bordetella* sp. PH22	Phenolic compounds-contaminated sediment	China	NCBI	JN171687.1	721	
*Bordetella* sp. VKRKCd3	Seashore surface sediment	India	NCBI	GQ262759.1	363	
**PLANT ORIGIN**						
***Bordetella*** **sp. CCBAU 10842**	Maize rhizosphere	China	NCBI	JF772555.1	1369	
***Bordetella*** **sp. R8–804**	*Jatropha curcas* L, plant root	Singapore	NCBI	JQ659985.1	1487	
***Bordetella*** **sp. R8–551**	*Jatropha curcas* L, plant root	Singapore	NCBI	JQ659951.1	1486	
***Bordetella*** **sp. S2–5-CL23**	velvetleaf seed	USA	NCBI	EU769148.1	1492	
*Bordetella* sp. S318(2010)	*M. sinensis* × giganteus internal stem tissue	Ireland	NCBI	HM102497.1	600	
*Bordetella* sp. Juv992	Lupine cluster roots	Switzerland	Weisskopf et al., 2011	JN590346.1	1302	
*Bordetella* sp. PnB 4	Pepper	India	NCBI	JQ886795.1	370	
*Bordetella* sp. RS-CIW-47	Maize rhizosphere	Pakistan	NCBI	KC430988.1	950	
**WATER ORGIN**						
***Bordetella*** **sp. MT-I2**	Industrial wastewater	Germany	Toups et al., 2010	EU727195.1	1526	1
***Bordetella*** **sp. MT-E1**	Industrial wastewater	Germany	Toups et al., 2010	EU727194.1	1525	1
***Bordetella*** **sp. TS-T34**	Lake water	China	NCBI	KC762319.1	1398	
***Bordetella*** **sp. CC-PW-55**	Surface seawater	Taiwan	NCBI	KF851340.1	1500	
***Bordetella*** **sp. 13.1 KSS**	Mineral oil-based metalworking fluid	Germany	Lodders and Kämpfer, 2012	HE575910.1	1398	
***Bordetella*** **sp. HT19**	Sulfur spring	India	NCBI	FJ969843.1	1404	
***Bordetella*** **sp. HF38**	River biofilms	China	NCBI	KR188914.1	1523	
***Bordetella*** **sp. HF72**	River biofilms	China	NCBI	KR188948.1	1523	
*Bordetella* sp. MMJ09	Distillery wastewater	China	NCBI	GU244378.1	813	
*Bordetella* sp. Sulf-8	Municipal wastewater	South Korea	Nisola et al., 2010	GU812430.1	1314	
*Bordetella* sp. IPJ1	Rusted iron pipe in freshwater lake	India	NCBI	HM593901.1	1100	

*In bold are the strains for which the length of the 16S ribosomal RNA sequence were at least 1376 bp, and were included in the phylogenetic tree construction*.

### 16S rRNA gene sequence clades are associated with particular environmental niches

**Figure 1:**
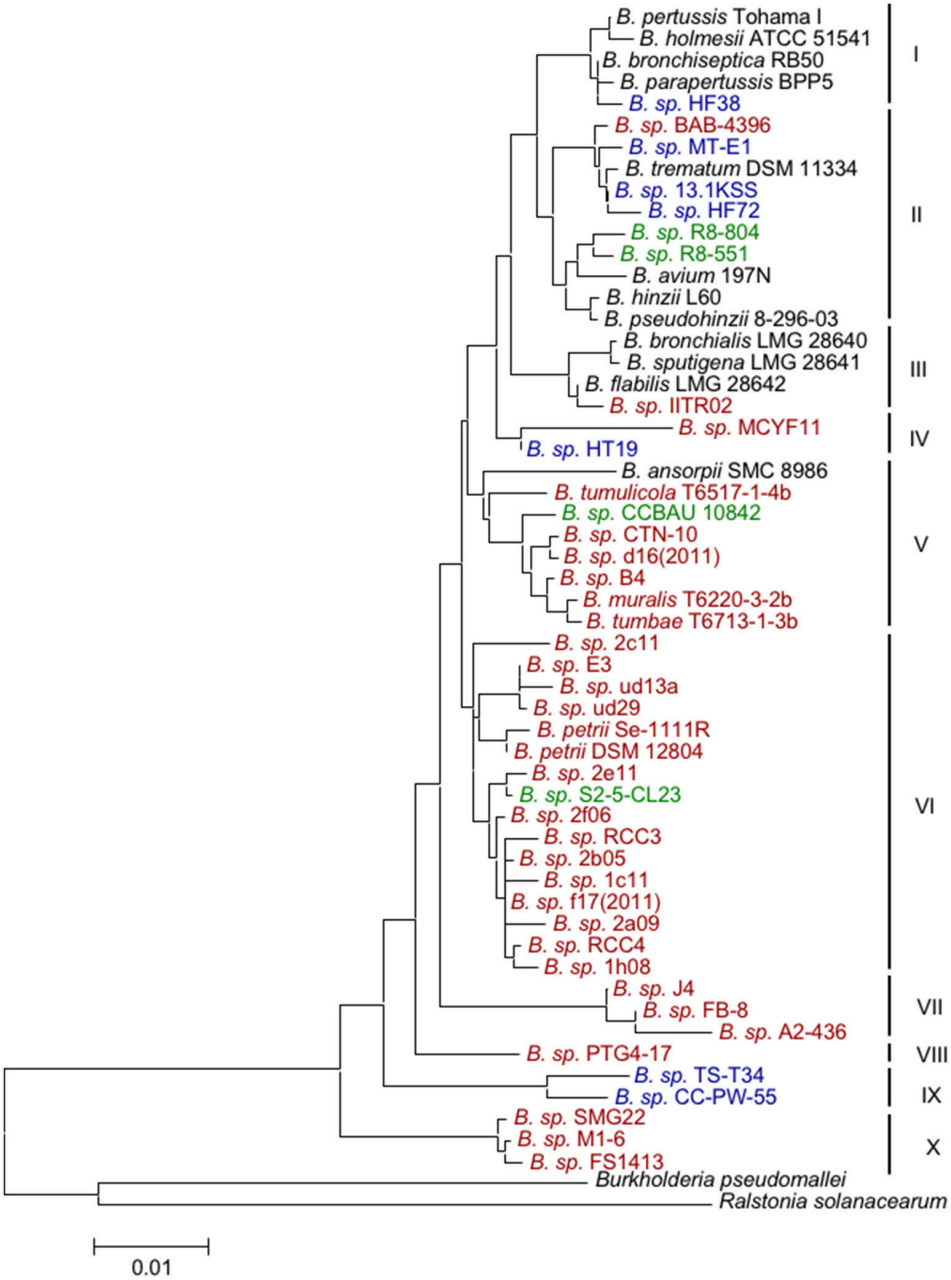
**Neighbor-Joining tree based on 16S rRNA gene sequences of animal-associated and environmental strains of ***Bordetella*****. The 52 near full-length sequences (1376 bp) formed 10 clades (I–X) of phylogenetically closely related *Bordetella* isolates/species recovered from soil (brown), water (blue), plants (green) and animals (black). The 16S rRNA gene sequences of the beta-proteobacteria *Burkholderia pseudomallei* and *Ralstonia solanacearum* were used as outgroups.

To relate the environmental isolates to known *Bordetella* species, we aligned the 16S rRNA gene sequences and constructed a Neighbor-joining tree using the Maximum-likelihood algorithm implemented in Mega (Tamura et al., 2007). Forty-eight sequences from environmental samples were of sufficient length and used for further analyses (Table 2). Of those, 36 originated from soil (27 haplotypes), eight from aquatic environments (7 haplotypes), and four from plants (4 haplotypes). The tree was rooted with sequences of *Burkholderia pseudomallei* and *Ralstonia solanacearum* as outgroups. The *Bordetella* sequences formed 10 distinct clusters (Figure 1). While most clusters contained at least one described species, such as *B. petrii* in cluster VI or *B. tumbae*/*B. muralis* in cluster V, several *Bordetella* sequences did not cluster with any described species but rather occupied distinct branches of the tree. These include the two isolates in cluster IV, the isolates from soil samples in clusters VII and X and strains *B*. sp. CC-PW-55 and *B*. sp. TS-T34 (cluster IX) isolated from surface sea water and lake water, respectively (Figure 1).

Superimposing the origin of the *Bordetella* spp. isolates revealed that most of the identified clusters were dominated by sequences of similar environmental/host origin. Thus, cluster I was composed of sequences of *B. holmesii* and the classical bordetellae (*B. bronchiseptica, B. parapertussis*, and *B. pertussis*), all of which were isolated from human and animal infection, but also contained *B*. sp. HT38 isolated from a river biofilm in China (Figure 1, Table 2). Cluster III contained sequences of species isolated from human respiratory specimen (*B. sputigena, B. bronchialis*, and *B. flabilis*) plus an isolate from soil in India. Other clusters either contained, or were dominated by, sequences of environmental origin such as cluster IV (water and soil), cluster V (soil), including the three species recovered from mural paintings *B. tumbae, B. tumulicola*, and *B. muralis*; but also *B*. sp. CCBAU from a maize rhizosphere and *B. ansorpii* from infection of an immunocompromised patient, and cluster VI (soil, including the environmental species *B. petrii*). The prominent exception to this pattern, cluster II, contained sequences from animal/human infection (*B. avium, B. hinzii, B. pseudohinzii*, and *B. trematum*) as well as from water (*B*. sp. MT-E1, *B*. sp. HF27), plant root (*B*. sp. R8–804, *B*. sp. R8–551), and soil samples (*B*. sp. BAB-4396). However, the other clusters were either dominated by animal-associated samples (clusters I and III) or samples of environmental origin (all other clusters).

If the genus *Bordetella* were of environmental origin, samples isolated from soil and water would be expected to be more diverse and would appear widespread across the tree. Indeed, environmental samples were present in all sequence clusters. In contrast, sequences from animal-associated samples were confined to four clusters, all of which also contained environmental isolates. Three of those four clusters formed a single super clade which originated from one of several clades among sequences from environmental isolates. In contrast, all clusters near the tree root exclusively contained environmental samples, but no animal associated samples (Figure 1). The phylogenetic analyses showed that the genetic diversity was significantly higher in sequences from environmental samples (Π_95_ = 2.02–2.13%) than in sequences from animal-associated samples (Π_95_ = 1.30–1.53%). The sequence of branching events within the phylogenetic tree is consistent with an environmental origin of *Bordetella* and subsequent adaptation of some lineages to animal hosts.

### *Bordetella bronchiseptica* and *Bordetella hinzii* are capable of growing in soil extract

**Figure 2:**
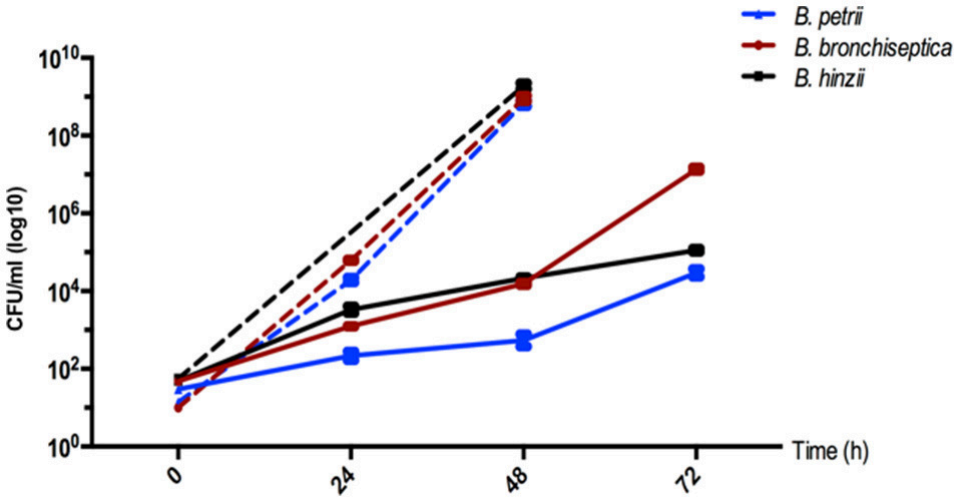
**Growth of ***B. bronchiseptica*** strain RB50, ***B. hinzii*** strain L60, and ***B. petrii*** strain DSMZ12804 in soil extract (solid lines) and in Stainer-Scholte medium (dashed lines)**. All three bacterial species efficiently grow in a sterile-filtered soil suspension suggesting that soil may represent an environmental niche for pathogenic *Bordetella* species.

Since most environmental *Bordetella* samples were recovered from soil (and water), we hypothesized that pathogenic, animal-associated species may have retained the ability to thrive in soil as an environmental niche. Therefore, we assessed the ability of *B. bronchiseptica* strain RB50, *B. hinzii* strain L60, and *B. petrii* strain DSMZ12804, to grow in a sterile, homogenized suspension made from soil. Instead of growing pathogenic bordetellae directly on solid soil, we prepared a soil suspension to extract possible nutrients but to avoid solid matter which allowed visual monitoring of bacterial growth and selection of appropriate sampling time points. All three isolates were cultured at room temperature (25°C) with shaking in either liquid soil extract or in Stainer-Scholte (SS) medium as a control. All three species grew fast in SS medium with doubling times of 1.8 ± 0.02 h (*B. bronchiseptica*), 1.9 ± 0.01 h (*B. hinzii*), and 1.9 ± 0.02 h (*B. petrii*), and reached the stationary phase prior to 48 h post-inoculation (Figure 2). As expected from an environmental bacterium, *B. petrii* strain DSMZ12804 thrived when inoculated into a soil extract, with a doubling time of 7.25 ± 0.24 h (Figure 2). Surprisingly, both *B. hinzii* strain L60 with a doubling time of 6.4 ± 0.09 h and *B. bronchiseptica* strain RB50 with a doubling time of 4.0 ± 0.04 h grew in the soil extract faster than *B. petrii*. Thus, all three species can grow efficiently at 25°C on filter-sterilized soil extract, even though the growth rate was slower than in Stainer-Scholte medium.

## Discussion

Bacteria of the genus *Bordetella* occupy remarkably diverse ecological niches, ranging from soil, water, and plants, to the respiratory tracts of a wide variety of animals including humans. Several environmental *Bordetella* strains were isolated from soils polluted with oil and oil derivatives (Table 2), including halogenated polycyclic hydrocarbons (Eriksson et al., 2003; Bianchi et al., 2005; Wang et al., 2007). Other strains were found in garden soil, compost, and various sediments suggesting these organisms are quite adaptable to diverse sites. The only sequenced and analyzed genome of an environmental isolate, *B. petrii* strain DSMZ 12804, revealed a possible genomic basis for substantial metabolic versatility (Gross et al., 2008). The genome encodes multiple auxiliary pathways for the utilization of a variety of nutrients, including pectate, numerous sugar derivatives from degraded plant products and various aromatic compounds. Five of the eight genomic islands that have been identified in this genome contain genes coding for enzymes for the metabolism of aromatic compounds, particularly clusters of genes encoding enzymes of the chlorocatechol pathway, including gene clusters that show high similarity to genes in a 1,2,4-trichlorobenzene-degrading *Pseudomonas* strain (Gross et al., 2008). The presence of multiple chlorocatechol gene clusters in addition to several different central pathways for aromatic metabolism may provide a competitive advantage for growth in contaminated environments.

Another striking feature of environmental *Bordetella* isolates is their resistance to heavy metals (Cavalca et al., 2010). Ten out of 52 soil samples (Table 2) were isolated from iron mines (e.g., *B*. sp. d16, *B*. sp. f17), from uranium mines (*B*. sp. FB-8, *B*. sp. A2–436), or from soil polluted with arsenic (e.g., *B*. sp. As3–3). Such remarkable metal tolerance is most likely conferred by heavy metal resistance systems. Indeed, the genome of *B. petrii* strain DSMZ 12804 contains several heavy metal resistance operons on a genomic island absent from the genomes of other sequenced bordetellae, whereas other strains contain different islands of genes. Ultimately, the presence of multiple heavy metal resistance systems may allow environmental *Bordetella* isolates to thrive in metal rich environments.

Most plant-associated *Bordetella* strains were recovered from roots (*B*. sp. R8–804, *B*. sp. R8–551, *B*. sp. Juv992) and the rhizosphere at the plant-soil interface (*B*. sp. CCBAU 10842). Thus, these isolates may in fact represent soil samples or, alternatively, may be involved in interactions with plants at the plant-soil interface. The resemblance between plant responses to bacterial virulence factors and the responses of mammalian immune cells (Berg et al., 2005) serve as evidence that bacteria-plant interactions may have paved the way for bacterial adaptation to animals. In this regard, plant-root isolates *B*. sp. R8–804 and *B*. sp. R8–551 from plant roots are closely related to bird pathogens, *B. hinzii* and *B. avium*, supporting the view that plants cells could serve as a “training ground” for environmental strains that eventually gain the ability to colonize animal hosts (Berg et al., 2005).

In addition to these plant root isolates, several other environmental isolates were also found to be very closely related to animal-associated pathogens (Figure 1). Interestingly, those strains were isolated from very diverse sources, namely (polluted) soil in India (*B*. sp. BAB-4396, *B*. sp. IITR02), industrial waste water (*B*. sp. MT-E1), and oil-based metal-working emulsion in Germany (*B*. sp. 13.1 KSS), as well as from river biofilms in China (*B*. sp. HF38 and *B*. sp. HF72). The two isolates from a river biofilm in China are of particular interest. The 16S rRNA sequence of one of those (strain HF72) showed 99.56% sequence similarity to that of the human pathogen *B. trematum* (6 SNPs). According to 16S rRNA gene sequence, the other isolate (*B*. sp. HF38) is even more closely related (99.78%, three SNPs) to the animal pathogen *B. bronchiseptica* strain RB50 and the human pathogen *B. parapertussis* strain 12822, which share an identical sequence in this gene. By this measure, isolate HF38 is as closely related to *B. bronchiseptica* strain RB50 and *B. parapertussis* strain 12822 as it is to *B. pertussis*. This exceptionally close phylogenetic relatedness makes several evolutionary scenarios conceivable. First, isolate *B*. sp. HF38 may be an environmental, non-pathogenic strain closely related to the animal/human pathogens among the classical bordetellae. Second, this isolate might be a descendant or relative of an ancestor of the classical bordetellae which later became pathogenic after acquisition of several virulence-associated factors, such as pertussis toxin, adenylate cyclase toxin, and dermonecrotic toxin. Third, this isolate may in fact represent a *B. bronchiseptica* or *B. parapertussis* strain that naturally survives and/or grows within an environmental reservoir. Although the classical bordetellae have not yet been isolated from outside a mammalian host, our results suggest that animal-pathogenic *Bordetella* species retain the ability to grow in soil as an environmental niche. This implies that *B. bronchiseptica* and other species might be found (at least transiently) in soil, for example at farms with suitable animal hosts such as cattle, pig, sheep and horse, or near dog kennels. Interestingly, even fastidious *B. pertussis* bacteria remained able to be cultured for up to 5 days when spread onto various hospital-setting surfaces such as fabrics, plastics, glass, and paper, and also in several infant foods (Ocklitz and Milleck, 1967). Fourth, *B*. sp. HF38 as well as other isolates from water and soil may be protected internally by a non-vertebrate host. For example, amoebae are known to host bacteria such as *Legionella pneumophila* (Molmeret et al., 2005), and amoeba-grown *L. pneumophila* exhibited radically increased resistance to harsh environmental conditions such as fluctuations in temperature, osmolarity, acidity, as well as to biocides that may facilitate bacterial survival and persistence in the environment (Barker et al., 1995; Abu Kwaik et al., 1997, 1998; Winiecka-Krusnell and Linder, 1999). Amoebae are ubiquitously found in most environments, and shared habitats between amoeba and *Bordetella* could be an important factor for the persistence of the bacteria. Indeed, our group has shown that the animal-adapted *B. bronchiseptica* is able to survive and multiply intracellularly in the trophozoites and sori of the amoeba *Dictyostelium discoideum* before being disseminated with the amoeba spores to novel geographical locations (Bendor et al., in revision). Thus, in addition to our recent data demonstrating that *B. bronchiseptica* can circulate and efficiently transmit amongst mammals, these data demonstrate that this species can also grow and disseminate efficiently in association with amoebae. These independent but interconnected *Bordetella* lifecycles allow for disease propagation, transmission, and re-emergence in the absence of an infected animal host.

Strains included in this study were identified as *Bordetella* spp. based on their 16S rRNA gene sequence. Currently, there are no data available regarding potential pathogenicity of these species. Whole genome sequencing will provide valuable insights into the evolution and ecology of environmental vs. animal-pathogenic bordetellae. Of special interest are environmental isolates closely related to animal pathogens, particularly isolate *B*. sp. HF38, and analysis of their genomes will reveal whether they are non-pathogenic relatives of known animal pathogens or if they in fact represent environmental reservoirs of *B. bronchiseptica* or *B. parapertussis*.

Finally, the majority of environmental *B*. sp. were recovered from soil samples indicating that soil could be the most frequent natural habitat of bordetellae. Indeed, sequences identified from soil samples were found in 8 of 10 sequence clusters, including samples from compost in cluster X at the root of the tree (Figure 1). The sequence of branching events within the phylogenetic tree, the significantly higher sequence diversity in samples from soil and water than in those from animals, as well as the preserved ability of animal pathogens to grow in soil, suggest an environmental, likely soil-based, origin of the genus *Bordetella*. Thus, similar to bacteria of the closely related genus *Achromobacter*, which are of environmental origin but also contain opportunistic pathogens (Li et al., 2013), *Bordetella* appears to be a bacterium of environmental origin that adapted and became pathogenic via the acquisition of factors mediating specific interactions with animal hosts.

## Author contributions

IHS, BL, and ETH conceived and designed the experiments. IHS and BL performed the experiments and analyzed the data. IHS, BL and ETH wrote the paper.

### Conflict of interest statement

The authors declare that the research was conducted in the absence of any commercial or financial relationships that could be construed as a potential conflict of interest.
